# GVS-12 attenuates non-alcoholic steatohepatitis by suppressing inflammatory responses *via* PPARγ/STAT3 signaling pathways†

**DOI:** 10.1039/c8ra10178g

**Published:** 2019-03-26

**Authors:** Yuhui Wang, Xiyang Zhang, Bo Yuan, Xi Lu, Dongxuan Zheng, Kefeng Zhang, Mingli Zhong, Xiaotian Xu, Xiaoqun Duan

**Affiliations:** Guangxi Colleges and Universities Key Laboratory of Pharmacology, Guilin Medical University 109 Huanchengbei Road Two Guilin 541004 China mufeng_91@163.com robortduan@163.com +86 773 2303451 +86 773 2303451

## Abstract

Non-alcoholic steatohepatitis (NASH), a type of fatty liver disease, is characterized by excessive inflammation and fat accumulation in the liver. Peroxisome proliferator-activated receptor γ (PPARγ) agonist rosiglitazone has great potential in protecting against the development of NASH. However, long-term usage of rosiglitazone probably leads to many adverse reactions. In this research, GVS-12 was designed and synthesized as a PPARγ agonist with high selectivity, evidenced by increasing the activity of the PPARγ reporter gene and promoting the mRNA expression of the PPARγ responsive gene cluster of differentiation 36 (CD36). It was noteworthy that GVS-12 could ameliorate dysfunction and lipid accumulation by down-regulating the mRNA expression of interleukin-1β (IL-1β), interleukin-6 (IL-6) and tumor necrosis factor-α (TNF-α) in the liver of high fat diet (HFD)-induced rats and palmitic acid (PA)-stimulated hepatocellular carcinoma G2 (HepG2) cells. Moreover, PPARγ siRNA (siPPARγ) markedly diminished GVS-12 induced the down-regulation of mRNA expression of IL-1β, IL-6 and TNF-α in PA-stimulated HepG2 cells. Additionally, GVS-12 could reduce the phosphorylation level of STAT3 and up-regulate the protein expression of a suppressor of cytokine signaling 3 (SOCS3), which could be reversed by siPPARγ. In detail, SOCS3 siRNA (siSOCS3) diminished the inhibitory effect of GVS-12 on the mRNA expression of IL-1β, IL-6 and TNF-α. In conclusion, GVS-12 suppressed the development of NASH by down-regulating the mRNA expression of IL-1β, IL-6 and TNF-α *via* PPARγ/STAT3 signaling pathways.

## Abstract 2

Non-alcoholic steatohepatitis (NASH), a type of fatty liver disease, is characterized by excessive inflammation and fat accumulation in the liver.

## Graphical abstract

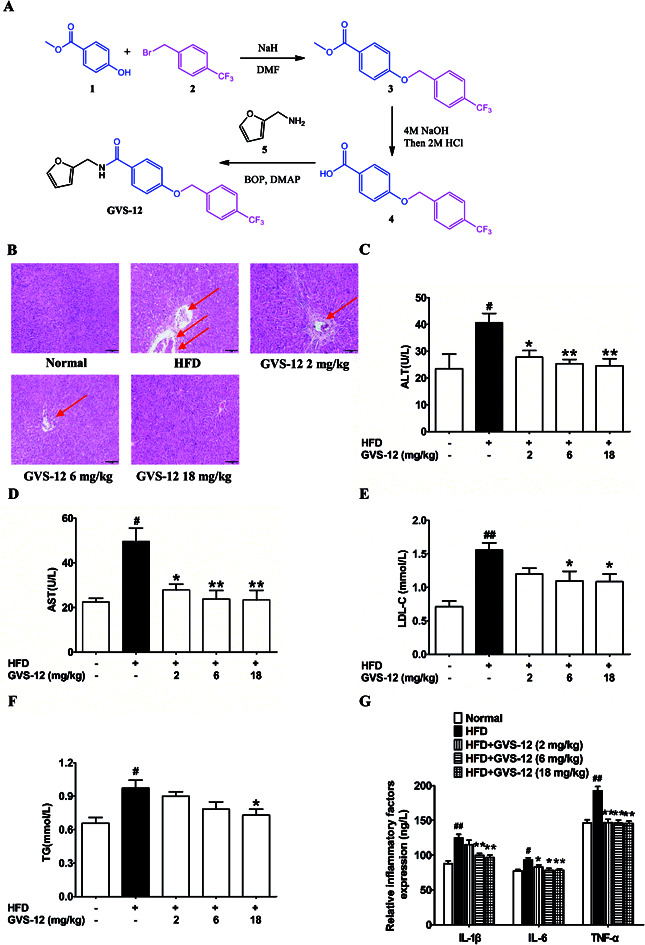

## 1. Introduction

Nonalcoholic steatohepatitis (NASH), as the most common cause of chronic liver disease, is characterized by fatty infiltration, fatty change, fat storage, necrosis and ballooning degeneration of hepatic cells accompanied by the infiltration of inflammatory cells.^1,2^ It affects millions of people worldwide. Hypothesis shows that the proinflammatory cytokines especially interleukin-1β (IL-1β), interleukin-6 (IL-6) and tumor necrosis factor-α (TNF-α) contribute to the occurrence and development of NASH, and consequently lead to liver cirrhosis and hepatocellular carcinoma.^3,4^ Therefore, down-regulating the expression of the proinflammatory cytokines might be a practical therapeutic strategy for NASH.

The key proinflammatory cytokines such as IL-1β, IL-6 and TNF-α could be regulated by many signal pathways. For example, the activation of p38 mitogen-activated protein kinase (MAPKs) signal pathways, nuclear factor kappa-light-chain-enhancer of activated B cells (NF-κB) signal pathways and signal transducer and activator of transcription 3 (STAT3) signal pathways could promote the production of the proinflammatory cytokines.^5^ In contrast, the inhibitors of these signal pathways could reduce the production of proinflammatory cytokines aforementioned. Accumulative evidence suggested that STAT3 signal pathway played a vital role in the production of proinflammatory cytokines in NASH.^6,7^ Therefore, the abrogation of aberrant STAT3 signaling pathways might decrease the production of the proinflammatory cytokines and inhibit the progress of NASH.

The thiazolidinediones, a class of heterocyclic compounds consisting of a five-membered C3NS ring, usually refers to a family of drugs used in the treatment of diabetes mellitus type 2.^8^ Rosiglitazone is an antidiabetic drug which activates peroxisome proliferator-activated receptor γ (PPARγ) in the thiazolidinedione class.^9^ It works as an insulin sensitizer through binding to the PPARγ in fat cells and making the cells more responsive to insulin. Increasing evidence showed that rosiglitazone could improve NASH through improving insulin resistance, lipid metabolism and reducing transaminase activity.^10,11^ The effect of rosiglitazone on the promotion of NASH was accomplished through inhibiting the inflammation responses *via* down-regulating the expression of proinflammatory cytokines.^12^ However, the long-term use of rosiglitazone could cause severe adverse reactions, such as increase of weight, retention of fluid and increase of the risk of heart disease.^13,14^ Therefore, to develop a new PPARγ agonist with high selectivity might be a strong strategy for NASH treatment.^15,16^ In combination of the advantage of structure features of rosiglitazone and SR1664, GVS-12 was designed and synthesized by our group. In the present study, we characterized the effects of GVS-12 on the inflammatory responses in high fat diet (HFD)-induced NASH rats and palmitic acid (PA)-induced hepatocellular carcinoma G2 (HepG2) cells. In depth, we explored the potential molecular mechanisms and target protein of GVS-12 with a focus on the PPARγ/STAT3 pathways.

## 2. Materials and methods

### 2.1 Reagents

**Fig. 1:**
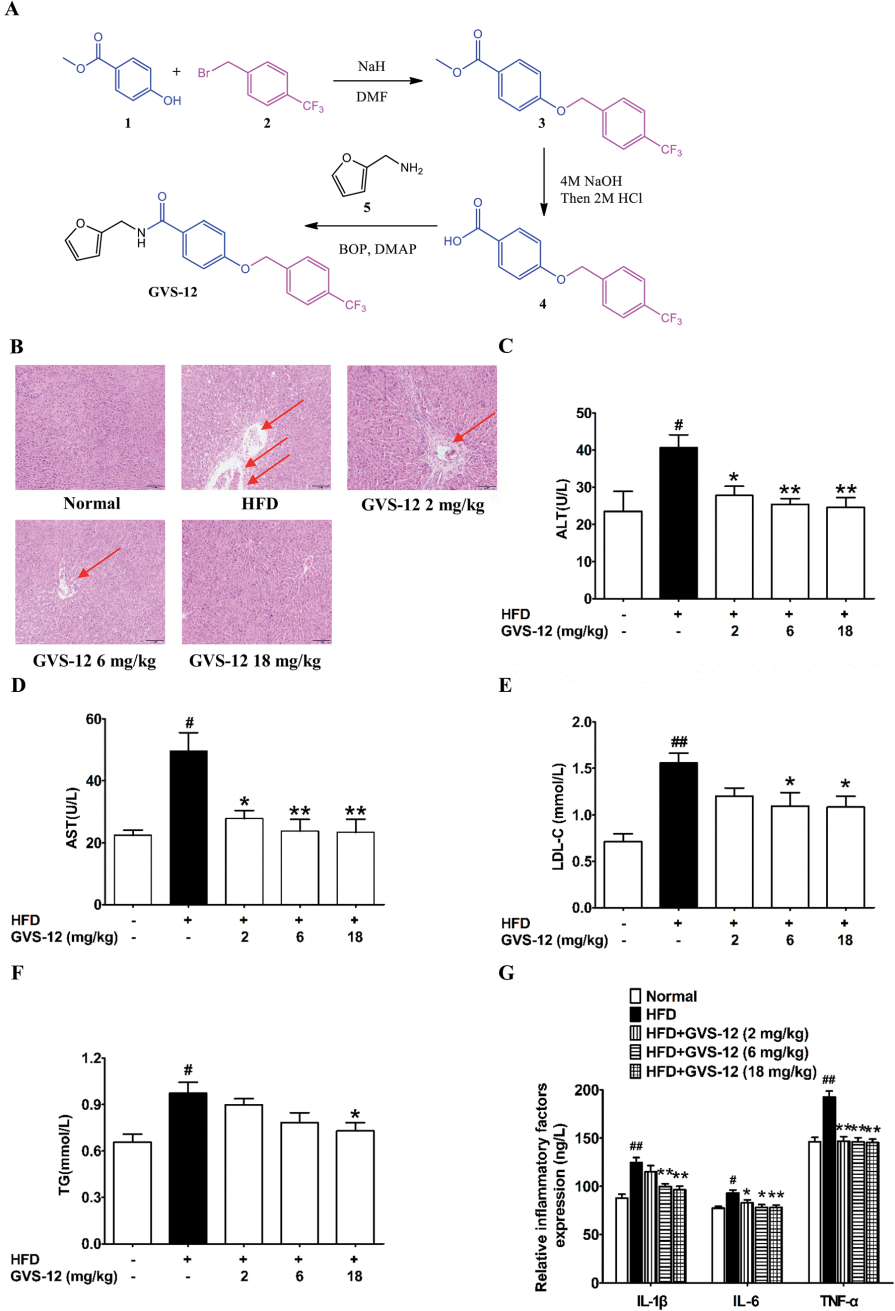
The synthesis route of GVS-12 and effect of GVS-12 on high fat diet (HFD)-induced rats. (A) The synthesis of GVS-12; (B) the pathological changes of the liver sections were detected by H&E staining, and the arrows indicated pathological changes of liver; (C–F) the serum level of liver function (ALT, AST, LDL-C and TG) was detected by biochemical quantitation kits; (G) the protein level of IL-1β, IL-6 and TNF-α in hepatic tissue was analyzed by ELISA. The data were expressed as the means ± SEM ^#^*p* < 0.05, ^##^*p* < 0.01 compared with the normal group; *p < 0.05, **p < 0.01, compared with the HFD group.

GVS-12 (C_20_H_16_F_3_NO_3_, MW: 375.346, purity ≥ 98%) was designed and synthesized by our team (Fig. 1A); LanthaScreen™ time-resolved fluorescence resonance energy transfer (TR-FRET) PPARγ competitive binding assay was purchased from Thermo Fisher Co., Ltd. (Thermo Fisher, MA, USA); fetal bovine serum (FBS) was obtained from PAA (Linz, Germany). TRIzol reagent was obtained from Invitrogen (Carlsbad, CA, USA). Rosiglitazone (a PPARγ agonist) was obtained from Sigma Co., Ltd. (St. Louis, MO, USA). STAT3, p-STAT3, suppressor of cytokine signaling 3 (SOCS3) and PPARγ antibodies were purchased from Abcam (Cambridge, UK); GAPDH monoclonal antibody was purchased from KangChen Bio-tech (Shanghai, China); horse radish peroxidase (HRP)-conjugated secondary antibodies were purchased from Abbkine (Redlands, USA). Other analytical reagent grade chemicals were obtained from Sinopharm Chemical Reagent Co., Ltd. (Nanjing, China).

### 2.2 Animals and experimental groups

Male 6 weeks-old SD rats were purchased from Cavens Laboratory Animals Co., Ltd. (Changzhou, China) and housed in 12 h light/12 h dark cycles in specific pathogen free laboratory animal rooms with unrestricted access to food and drinking water. This study was performed in strict accordance with the NIH guidelines for the care and use of laboratory animals (NIH publication no. 85-23 rev. 1985) and was approved by the Animal Ethics Committee of Guilin Medical University (Guilin, China). After an acclimatization period of 2 weeks, the rats were randomly divided into the following five groups (*n* = 6 per group), fed and treated as follows for 4 weeks: (1) normal group; (2) HFD group; (3) HFD + GVS-12 (2 mg kg^−1^) group; (4) HFD + GVS-12 (6 mg kg^−1^) group; (5) HFD + GVS-12 (18 mg kg^−1^) group. GVS-12 was orally administered seven times per week for the duration of the experiment. Thereafter, the rats were euthanized after a 12 h fast, and the liver tissues and blood samples were collected for analyses. Blood samples were acquired from the heart for serum isolation by centrifugation at 2000 × *g* for 10 min at 4 °C. The liver sections fixed with 4% paraformaldehyde were prepared for histological staining and the remained hepatic tissues were stored at −80 °C for molecular biological assays.

### 2.3 Enzyme-linked immuno sorbent assay (ELISA) and biochemical analysis

The level of hepatic triglyceride (TG), total cholesterol (TC) and low-density lipoprotein cholesterol (LDL-C) was detected by enzymatic colorimetric methods using commercially available kits (Nanjing Jiancheng Bioengineering institute, Nanjing, China) according to the manufacturer's protocol. Serum level of alanine aminotransferase (ALT) and aspartate transaminase (AST) was assessed by the microplate method (Nanjing Jiancheng Bioengineering institute) according to the manufacturer's instructions. Serum level of IL-1β, IL-6 and TNF-α was assessed by the ELISA analyses (Dakewe Biotech Co., Ltd) according to the manufacturer's instructions.

### 2.4 Liver histology analyses

Liver specimens were fixed in 4% paraformaldehyde, embedded in paraffin, and 4 μm-thick tissue sections were used for hematoxylin and eosin (H&E) staining. The non-alcoholic fatty liver disease (NAFLD) activity score (NAS) histological feature scoring system was utilized to assess liver lesion: steatosis (0–3), lobular inflammation (0–2), hepatocellular ballooning (0–2). Samples with scores > or = 5 were diagnosed as “NASH” and samples with scores < 3 were designated “not NASH”

### 2.5 Oil red O staining analysis

7 μm-thick frozen liver sections were fixed in 4% paraformaldehyde quickly and washed in deionized water for 3 times, and then blocked with oil red O solution for 10 min at room temperature, then washed with deionized water and stained with hematoxylin. In the end, tissue sections were sealed with glycerin gelatin and analyzed by light microscopy (Olympus, Tokyo, Japan).

### 2.6 Cell culture

Hepatocellular carcinoma G2 (HepG2) cells was obtained from American Type Culture Collection (ATCC, Manassas, VA, USA), and cultured in a humidified incubator at 37 °C under 5% CO_2_ atmospheric condition in corresponding medium supplemented with 10% FBS, 100 U mL^−1^ streptomycin and 100 U mL^−1^ penicillin.

### 2.7 Cell transfection

The PPARγ siRNA (siPPARγ) and SOCS3 siRNA (siSOCS3) were purchased from Jiman Biochemical Technology Co., Ltd (Shanghai, China). HepG2 cells were transfected with siPPARγ and siSOCS3 by using Lipofectamine 2000 (Invitrogen, Carlsbad, USA) according to manufacturer's instructions.

### 2.8 Quantitative real-time polymerase chain reaction (qPCR)

Palmitic acid (PA, 1 mM)-stimulated HepG2 cells and liver specimens were treated with GVS-12, and the total RNA was isolated by using TRIzol reagent according to the supplier's instructions. The cDNA was transcribed from RNA by using HiScript RT Super Mix (Vazyme, Nanjing, China), then analyzed for the expression of IL-1β, IL-6, TNF-α, SOCS3 and PPARγ by Ace Q-PCR SYBR Green Master Mix (Vazyme, Nanjing, China) with the help of MyiQ2 Detection System (Bio-Rad Laboratories, Hercules, USA).

### 2.9 Western blotting

PA-stimulated HepG2 cells and liver specimens were treated with GVS-12, and the total cell lysates were prepared by using nonidet P-40 (NP40) buffer (Beyotime, Nanjing, China). The equal concentration of protein lysate of all the samples was separated on 8% sodium dodecyl sulfate polyacrylamide gel electrophoresis (SDS-PAGE) gel and further transferred to polyvinylidene fluoride (PVDF) membranes. The membranes were blocked with 5% non-fat milk for 2 h, and then incubated with specific primary antibodies overnight at 4 °C. After being washed with tris buffered saline (TBS)-0.1% Tween, membranes were incubated with HRP-conjugated anti-mouse or anti-rabbit immunoglobulin G (IgG) secondary antibodies, and then visualized with electrochemiluminescence (ECL) reagent (Guge Biochemical Technology Co., Ltd., Wuhan, China).

### 2.10 Statistical analysis

The data was presented as the means ± standard error of measurement (SEM). Statistical analysis was performed by using one-way analysis of variance (ANOVA) followed by Tukey's test. *P* values less than 0.05 (*P* < 0.05) were accepted as a significant difference.

## 3. Results

### 3.1 The synthetic routes of GVS-12

As shown in Fig. 1A, (i) sodium hydride (NaH, 6.29 g, 12 mmol) was added in portions at 0 °C to a stirred solution of methyl 4-hydroxybenzoate 1 (1.52 g, 10 mmol) in dry *N*,*N*-dimethylformamide (DMF, 10 mL). After stirring for 30 min at 0 °C, 1-(bromomethyl)-4-(trifluoromethyl) benzene 2 (2.40 g, 10 mmol) was added and the mixture was then stirred at room temperature for 24 h. After this, the reaction mixture was cooled to ambient temperature, poured into H_2_O (100 mL) and extracted with ethyl acetate (EtOAc, 100 mL). The organic phase was dried over anhydrous sodium sulfate (Na_2_SO_4_). When the evaporation of the solvents was under reduced pressure, the crude product was purified on a silica gel column using EtOAc/petroleumether (1 : 9) to obtain the pure 3 (2.48 g, 80%) as a white solid. LC-MS (ESI): *m*/*z* 309 [M − H]^−^. (ii) A suspension of 3 (1.55 g, 5 mmol) and 5 mL sodium hydroxide (NaOH, 4 mol) in tetrahydrofuran (THF, 20 mL) was stirred at 50 °C until the consumption of the starting material. It was allowed to reach room temperature, hydrogen chloride (HCl, 2 mol) was added until the pH of the solution reached 3.0, and then extracted with EtOAc (50 mL) and washed by saturated solution of sodium chloride (NaCl). The combined organic phase was dried over anhydrous Na_2_SO_4_. After the evaporation of the solvents was under reduced pressure, the crude product was purified on silica gel column to afford the product 4 (1.33 g, 90%) as a white solid. LC-MS (ESI): *m*/*z* 295 [M − H]^−^. (iii) To a solution of product 4 (1.48 g, 5.0 mmol) in dry DMF (20 mL), dimethylaminopyridine (DMAP, 1.22 g, 10 mmol) and benzotriazol-1-yloxytris (dimethylamino) phosphonium hexafluorophosphate (BOP, 4.42 g, 10 mmol) were added respectively at 0 °C. After stirred for 0.5 h, furan-2-ylmethanamine 5 (0.49 g, 5.0 mmol) were added, the reaction was allowed to stir until all starting material disappeared. Then, the mixture was poured into a 10% aqueous solution of HCl. The aqueous phase was extracted with EtOAc for twice, and the combined organic layers were dried over anhydrous Na_2_SO_4_ and concentrated *in vacuo*. GVS-12 production (1.70 g, 98%) was purified on silica gel column as a white solid. ^1^H NMR (400 MHz, chloroform-d) *δ* 7.81–7.72 (m, 2H), 7.65 (d, *J* = 8.0 Hz, 2H), 7.58–7.52 (m, 2H), 7.37 (dd, *J* = 1.9, 0.9 Hz, 1H), 7.01–6.95 (m, 2H), 6.36–6.32 (m, 2H), 6.29 (dq, *J* = 3.4, 0.8 Hz, 1H), 5.16 (s, 2H), 4.66–4.59 (m, 2H). LC-MS (ESI):*m*/*z* 376 [M + H]^+^.

### 3.2 Effect of GVS-12 on the hepatic dysfunction and lipid accumulation in HFD-induced rats

As shown in Fig. 1B, after 4 weeks of HFD-diet feeding, the liver sections of the HFD model group showed vacuolated hepatocytes and severe micro- and macro-vesicular steatosis with infiltration of inflammatory cells as evidenced by H&E staining, all these above indicated that a proper NASH animal model had been established. It was noteworthy that GVS-12 (6, 18 mg kg^−1^) improved HFD diet-induced hepatic dysfunction as evidenced by reducing hepatocyte ballooning, lobular inflammation and steatosis in liver tissues. In addition, the HFD diet induced indicative damage of hepatocellular and increased the production of biochemical diagnostic markers such as ALT and AST in the serum of NASH rat, which were diminished by GVS-12 (2, 6, 18 mg kg^−1^) in a dose-dependent manner (Fig. 1C and D).

In order to elucidate the improvement of GVS-12 on hepatic steatosis, which is mainly manifested by lipid deposition and accumulation of lipid droplets, the contents of LDL-C and TG in the liver were assayed. In comparison with the normal rats, the production of LDL-C and TG was elevated in the liver of HFD-induced rats. However, the elevated production of LDL-C and TG was sharpened by GVS-12 (18 mg kg^−1^) (Fig. 1E and F).

An important pathological characteristic of NASH was hepatic inflammation, and HFD-diet previously showed to be efficacious in causing obvious hepatic inflammation. As shown in Fig. 1G, the production of IL-1β, IL-6 and TNF-α was increased in the liver of HFD rats compared with the normal group. GVS-12 (6, 18 mg kg^−1^) could reverse HFD-induced elevations of IL-1β, IL-6 and TNF-α in the liver. These results clearly showed the anti-steatosis effect of GVS-12 on HFD diet-induced NASH.

### 3.3 Effect of GVS-12 on the process of NASH in PA-induced HepG2 cells

**Fig. 2:**
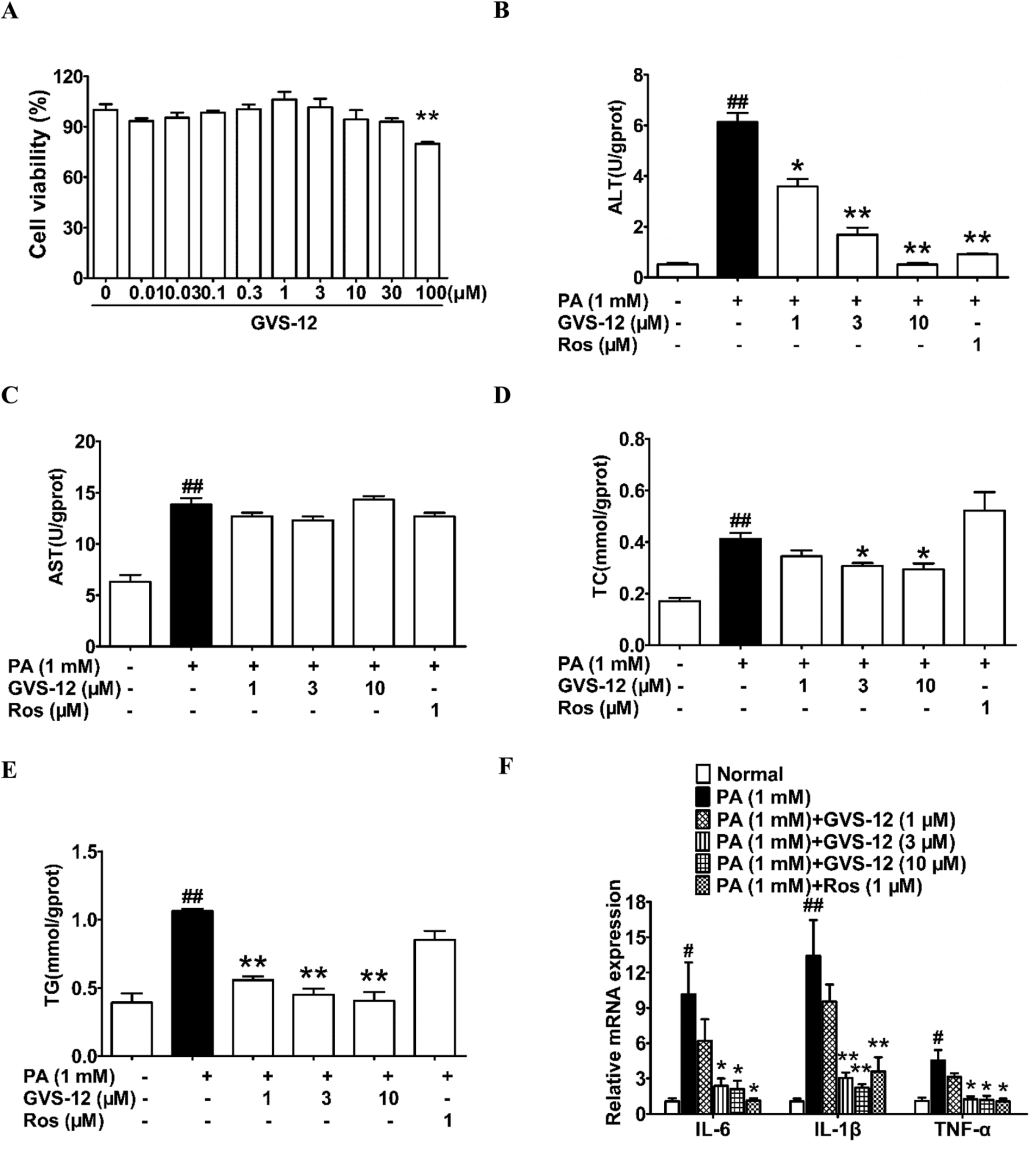
Effect of GVS-12 on NASH biomarkers in PA-induced HepG2 cells. (A) The effect of GVS-12 on HepG2 cell viability; HepG2 cells were induced by PA (1 mM) with or without GVS-12 (1, 3, 10 μM) and rosiglitazone (Ros, 1 μM). (B–E) The contents of ALT, AST, TC and TG were detected by biochemical quantitation kits; (F) the mRNA expression of IL-1β, IL-6 and TNF-α in PA-induced HepG2 cells was analyzed. The data were expressed as the means ± SEM of three independent experiments. ^#^*p* < 0.05, ^##^*p* < 0.01 *vs.* normal; *p < 0.05, **p < 0.01 *vs.* PA (1 mM).

MTT assay was used to determine the cell viability of the test compounds. The results showed that GVS-12 had little cytotoxicity on HepG2 cells at concentrations below 10 μM (Fig. 2A). Therefore, GVS-12 (1, 3, 10 μM) could be used for further experiments.

To disclose the protective mechanism of GVS-12 on NASH, we studied the effects of GVS-12 on the expression of lipid metabolism relative factors and proinflammatory cytokines in PA-stimulated HepG2 cells. As shown in Fig. 2B–E, the contents of ALT, AST, TC and TG were elevated in HepG2 cells treated with PA, and GVS-12 (3, 10 μM) reduced the contents of ALT, TC and TG with unobvious effect on the content of AST. Moreover, PA stimulation for 24 h resulted in a dramatical elevation of the mRNA expression of IL-1β, IL-6 and TNF-α in HepG2 cells, which were abrogated by GVS-12 (3, 10 μM) (Fig. 2F).

### 3.4 GVS-12 could activate PPARγ

**Fig. 3:**
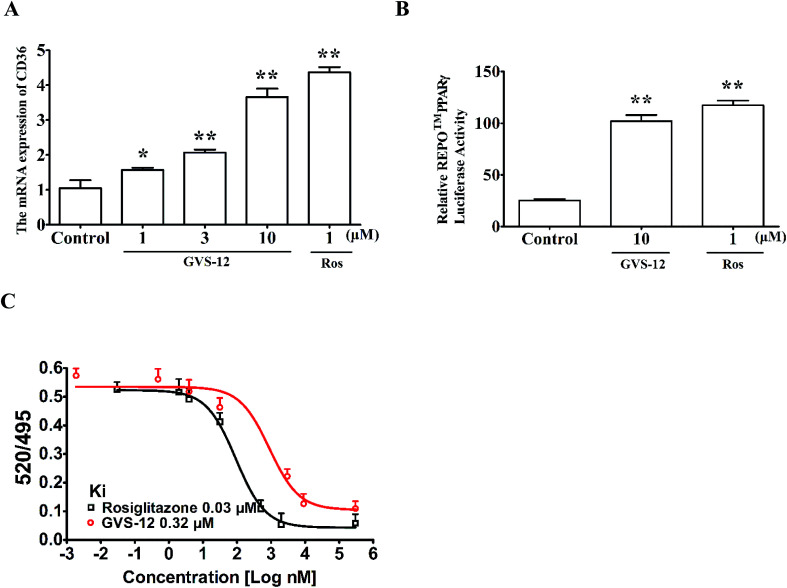
Effect of GVS-12 on the activation of PPARγ. (A) Effect of GVS-12 on the mRNA expression of CD36 in HepG2 cells. The cells were treated with GVS-12 (1, 3, 10 μM) and rosiglitazone (Ros, 1 μM) for 24 h. The mRNA expression of CD36 was detected using qPCR analysis. (B) Effect of GVS-12 on PPARγ reporter gene activity. HepG2 cells were transiently transfected with REPO™ PPARγ and then subjected to indicated treatments for 24 h. Cells were then harvested and assayed for luciferase activity. (C) Binding of GVS-12 to PPARγ-LBD in a competitive TR-FRET assay. Data were expressed as means ± SEM of three independent experiments. *p < 0.05 *vs.* control, **p < 0.01 *vs.* control.

Subsequently, we observed the effect of GVS-12 on the activation of PPARγ. As shown in Fig. 3A, the mRNA expression of cluster of differentiation 36 (CD36) (a target gene of PPARγ) was promoted by GVS-12 (1, 3, 10 μM) in HepG2 cells. Moreover, GVS-12 obviously enhanced PPARγ reporter gene activity in HepG2 cells (Fig. 3B). Furthermore, a TR-FRET assay demonstrated that GVS-12 bounded to PPARγ with a kinetic inhibition constant (*K*_i_) of 0.32 μM (Fig. 3C). These results suggested that GVS-12 could activate PPARγ.

### 3.5 A role of PPARγ in GVS-12-induced down-regulation of proinflammatory cytokines

**Fig. 4:**
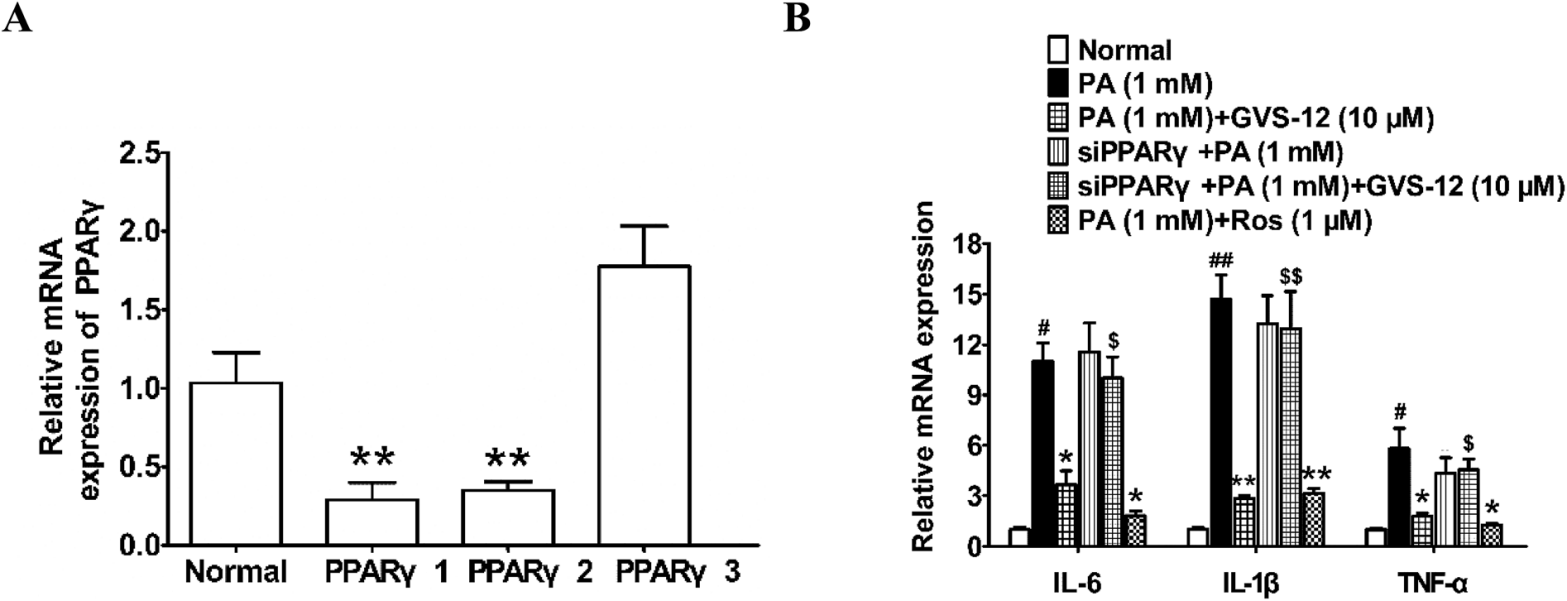
Effects of PPARγ on GVS-12 induced the down-regulation expression of IL-1β, IL-6 and TNF-α in PA-induced HepG2 cells. (A and B) Cells were transduced with siPPARγ, and then treated with GVS-12 (10 μM) or rosiglitazone (Ros, 1 μM) for 24 h before assay. The mRNA expression of IL-1β, IL-6 and TNF-α was analyzed by qPCR. The data were expressed as the means ± SEM of three independent experiments. ^#^*p* < 0.05, ^##^*p* < 0.01 *vs.* control, *p < 0.05, **p < 0.01 *vs.* PA (1 mM), ^$^*p* < 0.05, ^$$^*p* < 0.01 *vs.* GVS-12 (10 μM).

In order to identify the role of PPARγ in the down-regulated expression of IL-1β, IL-6 and TNF-α induced by GVS-12, PPARγ siRNA (siPPARγ) was used in PA-stimulated HepG2 cells. As shown in the Fig. 4A, PPARγ 1 showed stronger silence efficiency than PPARγ 2-3. Therefore, PPARγ 1 was used for further experiments. The results showed that siPPARγ could reverse the down-regulation effect of GVS-12 on the expression of IL-1β, IL-6 and TNF-α in HepG2 cells (Fig. 4B), indicating that GVS-12 suppressed the expression of IL-1β, IL-6 and TNF-α in a PPARγ-dependent manner.

### 3.6 A role of PPARγ in GVS-12-induced inhibition of STAT3 pathway

**Fig. 5:**
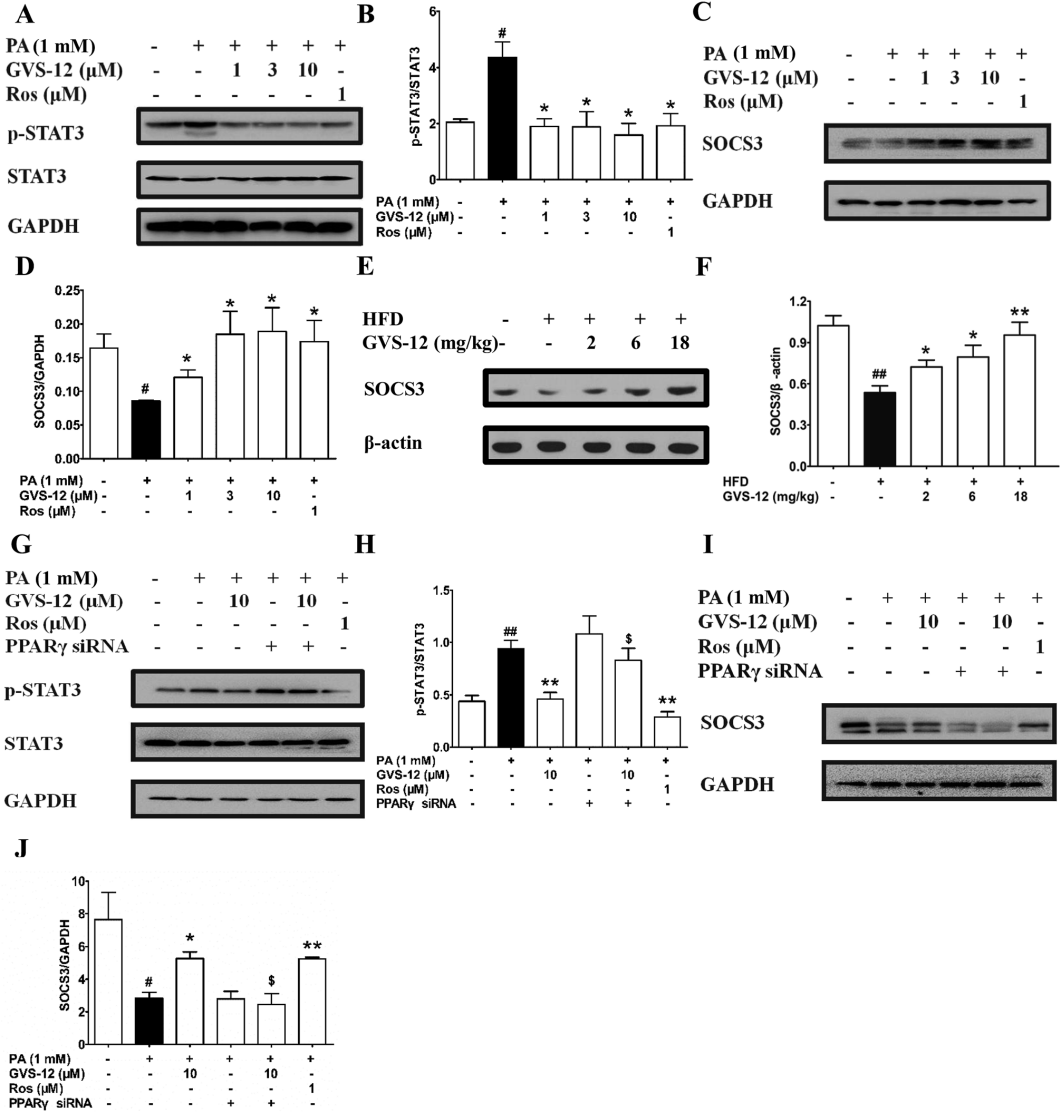
Effect of PPARγ on GVS-12 inhibited the activation of STAT3 in HFD-induced rats and PA-induced HepG2 cells. (A–D), HepG2 cells were incubated with PA (1 mM) in the accordance with GVS-12 (1, 3, 10 μM) or rosiglitazone (Ros, 1 μM) for the indicated internals. (A–D) The protein expression of STAT3, p-STAT3 and SOCS3 was detected by western blot; (E and F) rats were fed with HFD, and then treated with GVS-12 (2, 6, 18 mg kg^−1^). The expression of SOCS3 in the liver of HFD rats was assayed by western blot; (G–J) The protein expression of STAT3, p-STAT3 and SOCS3 in PA-induced HepG2 cells was detected by western blot. The data were expressed as the means ± SEM of three independent experiments. ^#^*p* < 0.05, ^##^*p* < 0.01 *vs.* control, *p < 0.05, **p < 0.01 *vs.* PA (1 mM), ^$^*p* < 0.05, ^$$^*p* < 0.01 *vs.* GVS-12 (10 μM).

To further explore the role of STAT3 in the inhibitory effect of GVS-12 on the expression of IL-1β, IL-6 and TNF-α, we observed the effect of GVS-12 on the activation of STAT3 in PA-stimulated HepG2 cells. Fig. 5A and B showed that the phosphorylation of STAT3 was inhibited by GVS-12 in PA-stimulated cells. Moreover, GVS-12 promoted the expression of SOCS3 (a suppressor of STAT3 signal transduction pathway) (Fig. 5C and D). Moreover, GVS-12 could promote the expression of SOCS3 in the liver of NASH rats (Fig. 5E and F). Of note, siPPARγ diminished the regulatory effect of GVS-12 on p-STAT3 and SOCS3, implying that GVS-12-induced the down-regulation of p-STAT3 level and the up-regulation of SOCS3 expression *via* activating PPARγ (Fig. 5G–J).

### 3.7 A role of SOCS3 in GVS-12-induced down-regulation of proinflammatory cytokines

**Fig. 6:**
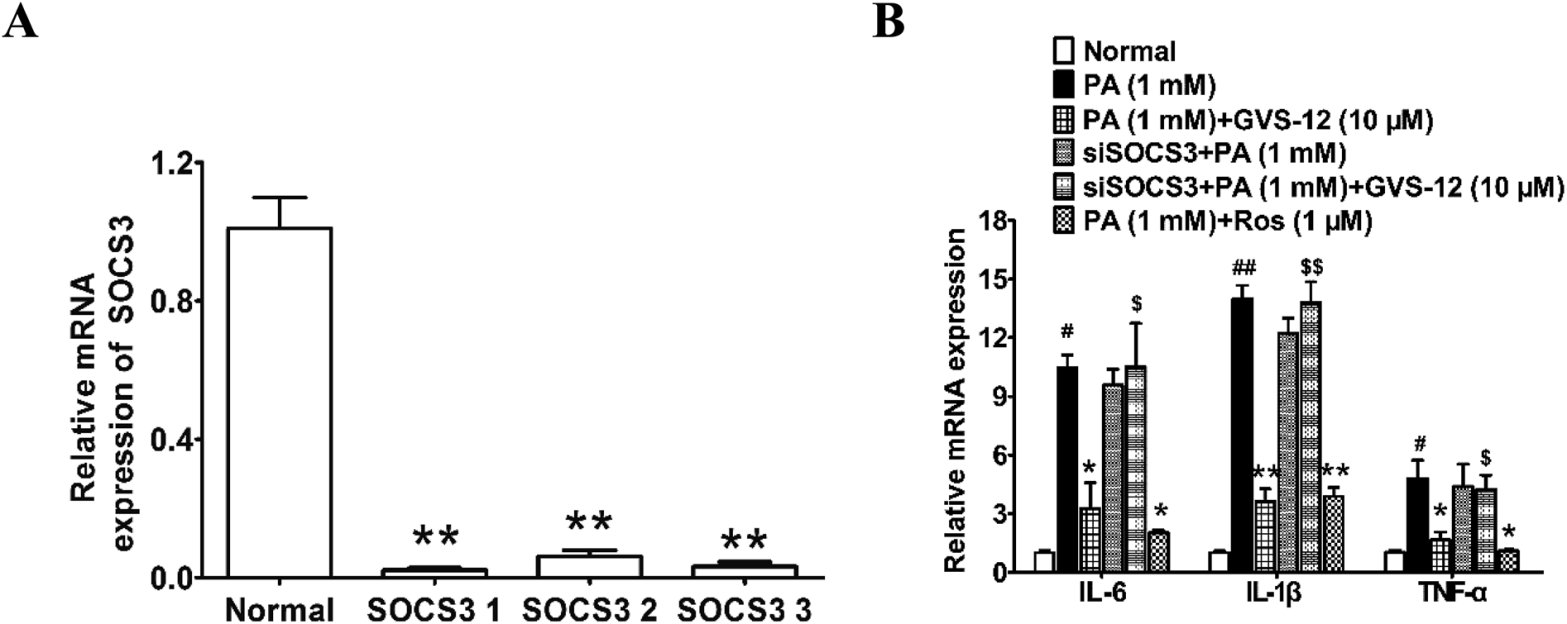
Effect of SOCS3 on GVS-12 induced the down-regulation expression of IL-1β, IL-6 and TNF-α in PA-induced HepG2 cells. (A and B) Cells were transduced with siSOCS3, and then treated with GVS-12 (10 μM) or rosiglitazone (Ros, 1 μM) for 24 h before assay. The mRNA expression of IL-1β, IL-6 and TNF-α was analyzed by qPCR. The data were expressed as the means ± SEM of three independent experiments. ^#^*p* < 0.05, ^##^*p* < 0.01 *vs.* control, *p < 0.05, **p < 0.01 *vs.* PA (1 mM), ^$^*p* < 0.05, ^$$^*p* < 0.01 *vs.* GVS-12 (10 μM).

To ascertain the relationship between the up-regulation of SOCS3 and down-regulation of IL-1β, IL-6 and TNF-α expression induced by GVS-12, PA-stimulated HepG2 cells were treated with GVS-12 in combination with or without SOCS3 siRNA (siSOCS3). As shown in the Fig. 6A, SOCS3 1 showed stronger silence efficiency than SOCS3 2-3. Therefore, SOCS3 1 was used for further experiments. The results showed that siSOCS3 diminished the down-regulation effect of GVS-12 on the mRNA expression of IL-1β, IL-6 and TNF-α (Fig. 6B), suggesting that SOCS3 played an important role in GVS-12-induced down-regulation of IL-1β, IL-6 and TNF-α expression.

## 4. Discussion

The primary hallmarks of NASH are fat storage, parenchyma hepatic cells fatty change, liver fatty infiltration, hepatic cells necrosis, ballooning degeneration, and inflammatory cells infiltration.^17,18^ Increasing evidence has shown that the infiltration of inflammatory cells in NASH mainly involves the secretion of inflammatory factors in the accumulating macrophage cells.^19–21^ It is worth noting that the secretion of inflammatory factors mainly includes the production of proinflammatory factors and the release of inflammatory factors. GVS-12, a new type of PPARγ, markedly down-regulated the mRNA expression of IL-1β, IL-6 and TNF-α in PA-induced HepG2 cells. Thus, we explored its potential mechanisms. The findings would deepen the anti-inflammatory effect of GVS-12 on the inflammatory cells infiltration of NASH, which could consecutively develop effective prevention or therapeutic strategies to combat NASH.

STAT3 is a member of the transcription and activation signal transduction family. Increasing evidence showed that the activation of STAT3 signaling pathway was critical in inflammatory responses, which promoted the production of pro-inflammatory factors such as IL-1β, IL-6 and TNF-α.^22,23^ The activation of STAT3 was closely correlated with fat storage, parenchyma hepatic cells fatty change, liver fatty infiltration, hepatic cells necrosis, ballooning degeneration and inflammatory cells infiltration in NASH.^24,25^ Therefore, it is probably of great therapeutic interest to develop effective medicine to suppress the activation of STAT3 in NASH patients. STAT3 activation could be inhibited by SOCS3, which was considered as a suppressor of STAT3 signaling pathway activation. The low expression or deficiency of SOCS3 results in the activation of STAT3 and secretion of inflammatory factors in NASH rats.^26,27^ Thus, up-regulating the expression of SOCS3 is a good method for inhibiting the activation of STAT3. Our study showed that GVS-12 obviously inhibited the mRNA expression of IL-1β, IL-6 and TNF-α in the liver of HFD-diet rats and PA-treated HepG2 cells. Moreover, GVS-12 down-regulated the expression of p-STAT3 and up-regulated the expression of SOCS3, indicating that STAT3 signaling pathway played a pivotal role in the inhibition of NASH treated with GVS-12.

PPARγ, a ligand-dependent transcription factor that can regulate fatty acid storage and glucose metabolism, plays a vital role in NASH.^28,29^ Increasing evidence has indicated that PPARγ activation might inhibit the inflammatory responses in the liver of NASH rats.^30^ There is growing evidence affirmed that PPARγ agonist rosiglitazone could alleviate hepatocyte steatosis through inhibiting the activation of STAT3 pathway.^31^ However, the long-term use of rosiglitazone might cause adverse reactions such as weight gain, fluid retention, and an increase in the risk of heart disease. Therefore, developing a new type of PPARγ agonist with high selectivity might be a better strategy for NASH treatment. Combining the superiority of rosiglitazone and SR1664, GVS-12 was designed and synthesized as a PPARγ agonist by our team. We found that GVS-12 was a PPARγ modulator with low toxicity (ESI†), and PPARγ was involved in GVS-12-mediated inhibition of STAT3 phosphorylation and consequent inhibition of inflammatory responses. The result showed that GVS-12 could promote the expression of CD36 in HepG2 cells. The TR-FRET assay further demonstrated that GVS-12 had the ability to bind PPAR with a binding constant of 0.32 μM. Moreover, GVS-12 was shown to increase the reporter gene activity in a PPAR-dependent manner. In summary, GVS-12 is a PPARγ regulator, and it could activate PPARγ. The effect and mechanism of GVS-12 on NASH might be as same as PPARγ agonists.^32–34^ We assumed that *p*-hydroxybenzoic acid might be a metabolic product of GVS-12. And it would be evaluated in a series of preclinical efficacy pharmacology, safety pharmacology, pharmacokinetic/toxicokinetic, and drug metabolism studies for further experiments.

## 5. Conclusion

In conclusion, GVS-12 alleviated the NASH process through inhibiting the expression of IL-1β, IL-6 and TNF-α both *in vivo* and *in vitro*. Moreover, it mainly functioned by regulating the SOSC3/STAT3 pathway *via* activating PPARγ.

## Conflicts of interest

The authors have no financial conflicts of interest.

## Supplementary Material

RA-009-C8RA10178G-s001
