# Supplementary material for: GVS-12 attenuates non-alcoholic steatohepatitis by suppressing inflammatory responses via PPARγ/STAT3 signaling pathways
Source: RSC Adv. 2019 Mar 26;9(17):9555–64. doi: 10.1039/c8ra10178g (PMC9062151; doi:10.1039/c8ra10178g)
Supplement: RA-009-C8RA10178G-s001 [file RA-009-C8RA10178G-s001.pdf]

Electronic Supplementary Material (ESI) for RSC Advances.
This journal is © The Royal Society of Chemistry 2019

As shown in Figure. 1, rosiglitazone (1 μM) powerfully stimulated adipocyte differentiation at the concentration of 1 μM by intensive Oil-red O staining of cytoplasmic fat droplets. In the control group, hardly any red cells were visible in the untreated cells. Interestingly, when 3T3-L1 cells were treated with 10 μM of GVS-12, very few cells were stained.

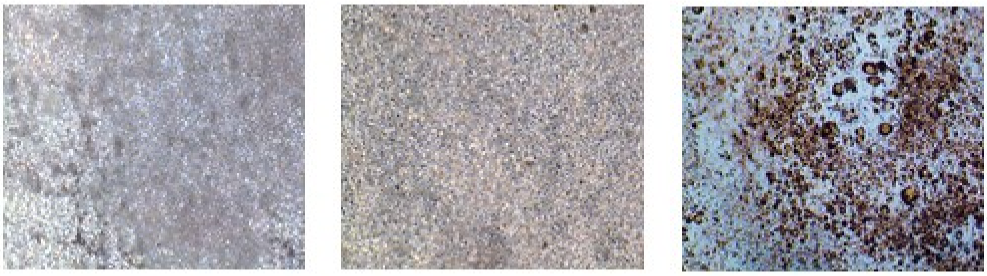

Control **GVS-12 (10 μM)** **Rosiglitazone (1 μM)**

Figure. 1 Lipid accumulation in differentiated 3T3-L1 cells treated with rosiglitazone or GVS-12 by Oil Red O staining. 3T3-L1 fibroblast cells were induced by 1 μM of dexamethasone, 0.5 mM of isobutylmethylxanthine, and 850 nM of insulin for 48 h and cells were switched to maintenance medium containing 850 nM of insulin for 6 days.
